# Visualization of Brain Activity in a Neuropathic Pain Model Using Quantitative Activity-Dependent Manganese Magnetic Resonance Imaging

**DOI:** 10.3389/fncir.2019.00074

**Published:** 2019-11-26

**Authors:** Chihiro Inami, Hiroki Tanihira, Satomi Kikuta, Osamu Ogasawara, Kazuya Sobue, Kazuhiko Kume, Makoto Osanai, Masahiro Ohsawa

**Affiliations:** ^1^Department of Neuropharmacology, Graduate School of Pharmaceutical Sciences, Nagoya City University, Nagoya, Japan; ^2^Graduate School of Medicine, Tohoku University, Sendai, Japan; ^3^Systems Neuroscience Section, Primate Research Institute, Kyoto University, Inuyama, Japan; ^4^Department of Anesthesiology, Graduate School of Medicine, Nagoya City University, Nagoya, Japan; ^5^Graduate School of Biomedical Engineering, Tohoku University, Sendai, Japan; ^6^Division of Health Sciences, Department of Medical Physics and Engineering, Graduate School of Medicine, Osaka University, Suita, Japan

**Keywords:** neuropathic pain, MRI, manganese, somatosensory abnormality, emotion

## Abstract

Human brain imaging studies have revealed several regions that are activated in patients with chronic pain. In rodent brains, functional changes due to chronic pain have not been fully elucidated, as brain imaging techniques such as functional magnetic resonance imaging and positron emission tomography (PET) require the use of anesthesia to suppress movement. Consequently, conclusions derived from existing imaging studies in rodents may not accurately reflect brain activity under awake conditions. In this study, we used quantitative activation-induced manganese-enhanced magnetic resonance imaging to directly capture the previous brain activity of awake mice. We also observed and quantified the brain activity of the spared nerve injury (SNI) neuropathic pain model during awake conditions. SNI-operated mice exhibited a robust decrease of mechanical nociceptive threshold 14 days after nerve injury. Imaging on SNI-operated mice revealed increased neural activity in the limbic system and secondary somatosensory, sensory-motor, piriform, and insular cortex. We present the first study demonstrating a direct measurement of awake neural activity in a neuropathic pain mouse model.

## Introduction

Chronic pain sensitizes against somatosensory stimuli and induces anxiety and depression in patients (Sah et al., 2003). These physical and psychological changes are caused by chronic alterations in brain activity. Neuropathic pain is caused by abnormal neural excitation elicited by conditions such as nerve injury, diabetes mellitus, herpes simplex virus infection, and human immunodeficiency virus infection. Since neuropathic pain is resistant against many analgesics including opioids, there are very few therapeutic options. It is established that neuropathic pain results in neuroplasticity that can be observed in several brain regions (Jaggi and Singh, 2011). This activation complements the fact that pain is a subjective and multidimensional experiences constructed by a combination of sensory, emotional, and cognitive experiences (Tracey, 2010).

An obvious difference between humans and rodents is the communication methods regarding their physical conditions. Human patients can verbally report pain, while animals may exhibit pain through behavioral changes. Currently, assessing persistent pain using animal models is difficult because the animals often do not exhibit any pain-related behaviors despite experiencing pain. Efforts to measure persistent pain in rodents include the use of ultrasonic vocalizations, facial expressions, altered locomotion, and altered sleep patterns (Jourdan et al., 2002; Wallace et al., 2005; Langford et al., 2010; Mogil et al., 2010; Urban et al., 2011). As these measures of persistent pain did not yield consistent and conclusive results, assessment of neuropathic pain in rodents typically relies on behavioral measures of mechanical and/or thermal thresholds (D’Amour and Smith, 1941; Le Bars et al., 2001). These types of measurements may be difficult to use as interpretations of neuropathic pain in human patients (Gottrup et al., 1998; Backonja and Stacey, 2004; Baron et al., 2009). Based on these reports, it is clear that adequate assessment methods for chronic pain in rodents are still lacking (Borsook et al., 2011).

In humans, imaging studies have revealed brain regions activated by chronic pain, including the primary somatosensory, secondary somatosensory, prefrontal, insular, anterior cingulate cortices and the thalamus (Baliki et al., 2006; Howard et al., 2012). *In vivo* brain imaging in rodents has revealed activation in homologous brain regions in response to acute noxious stimuli (Borsook et al., 2011; Thompson and Bushnell, 2012). Using positron emission tomography (PET) with the metabolic tracer [^18^F]fluorodeoxyglucose (FDG) on a rodent model of neuropathic pain, increased brain activity was observed in the somatosensory cortex, a change not seen in rodents assessed under general anesthesia (Thompson et al., 2014). Increased activation was also observed in the prefrontal-limbic-brainstem areas in an awake rat model of neuropathic pain using micro-PET with [^18^F]FDG (Kim et al., 2014). However, direct measurement of neural activity in awake resting-state rodents has not been successful.

Activation-induced manganese-enhanced magnetic resonance imaging (AIM-MRI) is a method for examining brain activation patterns in rodents (Lin and Koretsky, 1997; Aoki et al., 2004). Manganese ion (Mn^2+^) is an excellent MRI-detectable T_1_ contrast agent (Duyn and Koretsky, 2008; Silva and Bock, 2008), because it shortens the longitudinal relaxation time (T_1_) of proton (H^+^). Mn^2+^ can pass through voltage-gated calcium channels (Nelson, 1986; Narita et al., 1990), and accumulates in active neurons (Kikuta et al., 2015). The accumulated Mn^2+^ in neurons is maintained for more than 48 h (Yu et al., 2005; Tanihira et al., unpublished observation). Kikuta et al. (2015) showed that the amount of accumulated Mn^2+^ is linearly correlated with neuronal activity. It is important to note that Mn^2+^ is taken up into brain parenchyma after intraperitoneal (i.p.) administration of MnCl_2_ (Kikuta et al., 2015; Tanihira et al., unpublished observation). Since the slow and uniform diffusion of Mn^2+^ into the entire extracellular brain space enables recording of the history of neuronal activity in awake, freely moving animals (Van der Linden et al., 2007), AIM-MRI can map the activated brain regions after MnCl_2_ administration (Duyn and Koretsky, 2008; Silva and Bock, 2008; Tambalo et al., 2009; Koretsky, 2012; Kikuta et al., 2015).

The present study investigates the steady-state brain activity of neuropathic pain in awake mice. AIM-MRI with quantitative T_1_ measurement (qAIM-MRI), quantifying T_1_ values (Tambalo et al., 2009; Kikuta et al., 2015), was used on a spared nerve injury (SNI) model of neuropathic pain (Yamamoto et al., 2016). We found that steady-state brain activity in an awake neuropathic pain mouse model showed increased activity of the prefrontal-limbic-basal ganglia circuit. To our knowledge, this study is first to directly measure neural activity in animals with neuropathic pain under awake conditions.

## Materials and Methods

### Ethical Issues

All animal studies were approved by the Animal Care Committee of the Graduate School of Pharmaceutical Sciences, Nagoya City University and by the Tohoku University Committee for Animal Experiments. Experiments were conducted in accordance to the guidelines of the National Institute of Health and the Japanese Pharmacological Society.

### Animals

We used male C57BL/6J mice (6 weeks old; CLEA Japan, Shizuoka, Japan). All mice were housed in a room maintained at 23 ± 2°C with an alternating 12-h light-dark cycle and had *ad libitum* access to food and water. Each mouse was used only once.

### Spared Nerve Injury Model

The surgical procedure for producing the SNI model was originally described by Decosterd and Woolf (2000). Animals were anesthetized with isoflurane (4% for induction, 2% for maintenance). An incision was made in the skin on the lateral surface of the left thigh, followed by a section through the biceps femoris muscle to expose the sciatic nerves. The common peroneal and tibial nerves were then tightly ligated with 8–0 silk suture, sectioned distal to the ligation, and 1 mm of the distal nerve stump was cut. The sural nerve was left intact, taking care not to stretch it. Sham-operated controls were subjected to exposure of the sciatic nerve and its branches without any lesions.

### Tactile Allodynia

Tactile allodynia was evaluated by measuring the hind paw withdrawal responses to von Frey filaments (Touch-test^®^ sensory Evaluators; North Coast Medical, Gilroy, CA, United States), with a pressure ranging from 0.02 to 1.4 g (0.02, 0.04, 0.07, 0.16, 0.4, 0.6, 1.0, and 1.4 g). Mice were placed in cages with wire-mesh floors. The 50% likelihood of a paw withdrawal response (50% threshold) was determined using the up-down method (Dixon, 1965). Testing was initiated with the 0.16 g filament, and each filament was applied perpendicularly within the area innervated by the sural nerve on the lateral plantar surface of the left hind paw with sufficient force to cause slight bending of the filament for a duration of about 3 s (Decosterd and Woolf, 2000). If a positive response (lifting of the hind paw) was elicited, the next weakest filament was used. If a negative response (absence of hind paw withdrawal) was elicited, the next strongest filament was used. We continued until four measurements had been obtained after an initial change in behavior or until four consecutive positive (0.02 g) or five negative (1.4 g) scores had been obtained. The resulting scores were used to calculate the 50% threshold (Chaplan et al., 1994).

### Quantitative Activation-Induced, Manganese-Enhanced Magnetic Resonance Imaging, and MR Image Analysis

Two weeks after SNI surgery, mice in both SNI and sham-operated groups were injected with MnCl_2_ solution (0.2 mmol/kg in saline, intraperitoneally) twice at 24-h intervals (Kikuta et al., 2015). The methods for MRI acquisition were described previously (Kikuta et al., 2015). Brief methods are as follows: MRI acquisition was conducted 48 h after the second MnCl_2_ administration. The animals were anesthetized with 1–2% isoflurane (Mylan). Body temperature was maintained by circulation of heated water under the body. For T_1_ measurement of the brain, rapid acquisition with relaxation enhancement (RARE), and variable repetition time (RARE-VTR) pulse sequence with 7 TR values (450, 600, 900, 1500, 2500, 4500, and 7500 ms) was used with effective echo time (TEeff) = 8.1 ms, matrix size = 128 × 128, field-of-view (FOV) = 1.6 cm^2^ × 1.6 cm^2^, slice thickness = 0.5 mm, and number of slices = 20. Multislice, fast spin-echo T_2_-weighted images (RARE, TEeff = 22 ms, TR = 2500 ms) were acquired and used to co-register images to the mouse brain template acquired in advance (Kikuta et al., 2015). All measurements were carried out in a 9.4 T MRI scanner (AV400WB, Bruker) equipped with a 45 G/cm gradient and a 38 mm ^1^H volume coil (Bruker). The total time required to obtain all magnetic resonance images was approximately 45 min.

MRI analysis methods were also described previously (Kikuta et al., 2015). Brief methods are as follows: after spatial filtering, parametric T_1_ maps were calculated pixel-by-pixel by fitting with the following equation using Para Vision 5.1 software (Bruker BioSpin).

SI(TR)=A-Bexp(-TR/T)1,

where SI is signal intensity in each pixel.

The T_2_-weighted images were registered to the previously described T_2_-weighted template image (Kikuta et al., 2015), and the T_1_ maps were co-registered simultaneously using SPM12 software (Wellcome Trust Center for Neuroimaging, University College of London). Using the mouse brain atlas (Lein et al., 2007) Allen Institute for Brain Science. Allen Mouse Brain Atlas^1^) registered to the T_2_-weighted template image (Kikuta et al., 2015), we could then identify brain regions by querying structures from the brain atlas. An unpaired Student’s *t*-test was used to determine which T_1_ voxels decreased or increased in the SNI group compared with the sham group using SPM12. A parametric map of voxels with statistically significant changes in T_1_ was created and overlaid on the T_2_-weighted template image.

ROIs were set in the area where significant changes in T_1_ values were observed in SPM analysis. The ROI size was 3 × 3 × 3 voxels. Average T_1_ values in ROIs were calculated for statistical analysis.

### Statistical Analyses

Statistical analysis was performed using R, MATLAB (Mathworks), and SPM12 software. For the statistical parametric mapping (SPM) analysis, statistical significance (*p* < 0.025) was assessed by the unpaired Student’s *t*-test using SPM12 software. To ascertain the variability of T_1_ values within each group, we employed the Mann-Whitney *U*-test, and if a significant difference (*p* < 0.05) was detected, we confirmed the significance of the mean T_1_ values by bootstrapping within a 95% confidence interval after 1,000 randomizations. All data are presented as mean ± standard error of mean.

## Results

### Spared Nerve Injury Model Mice Exhibited Tactile Allodynia at 14 Days After Surgery

**FIGURE 1:**
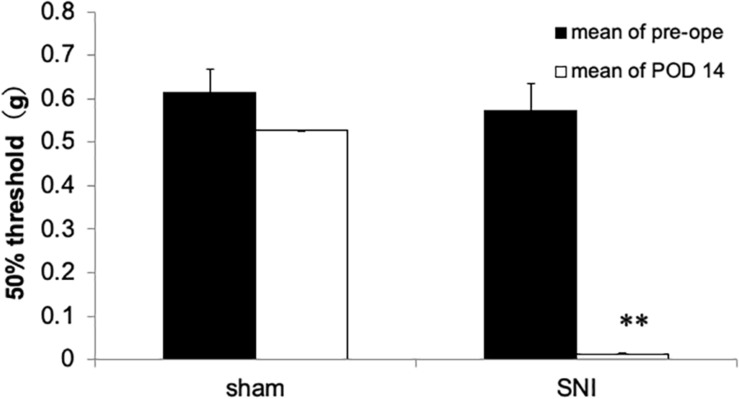
Mechanical nociceptive threshold of the hind paw in spared nerve injury (SNI) and sham-operated mice 14 days after nerve ligation. The SNI group exhibited decreased mechanical nociceptive threshold compared with the sham-operated group. Each column represents the mean ± SEM from 5 (sham group) and 8 (SNI group). Where error bars are not visible, they are smaller than the symbol. ^∗∗^*p* < 0.01 vs. before surgery (pre, Student’s *t*-test).

Fourteen days after SNI surgery, mice exhibited a prominent decrease of mechanical nociceptive threshold in the injured paw (Figure 1). In contrast, sham-operated mice did not exhibit the reduction of mechanical nociceptive threshold (Figure 1).

### qAIM-MRI Showed Increased Activation of Several Brain Region in SNI Model Mice

**FIGURE 2:**
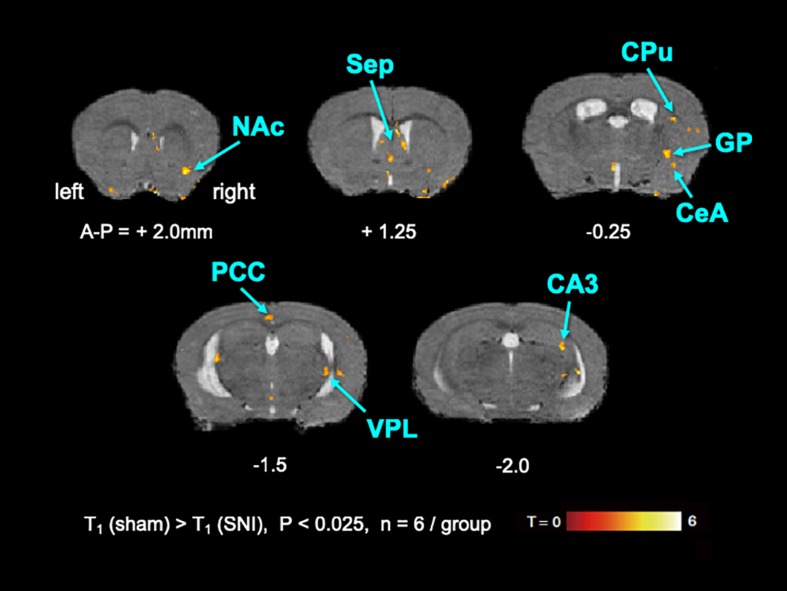
The active regions within the limbic structure in SNI mice compared with sham mice analyzed by AIM-MRI. Regions with significant shortening of T1 in SNI mice are indicated by the pseudo-colored regions over the T2-enhanced brain image template in coronal planes (*n* = 6 for sham, *n* = 6 for SNI). The active regions within the limbic structure are defined in text: nucleus accumbens (NAc), caudate-putamen (CPu), globus pallidus (GP), and ventral posterolateral nucleus of thalamus (VPL). The distance from bregma in mm is shown at the bottom. T, *t*-value; P, *p*-values.

**FIGURE 3:**
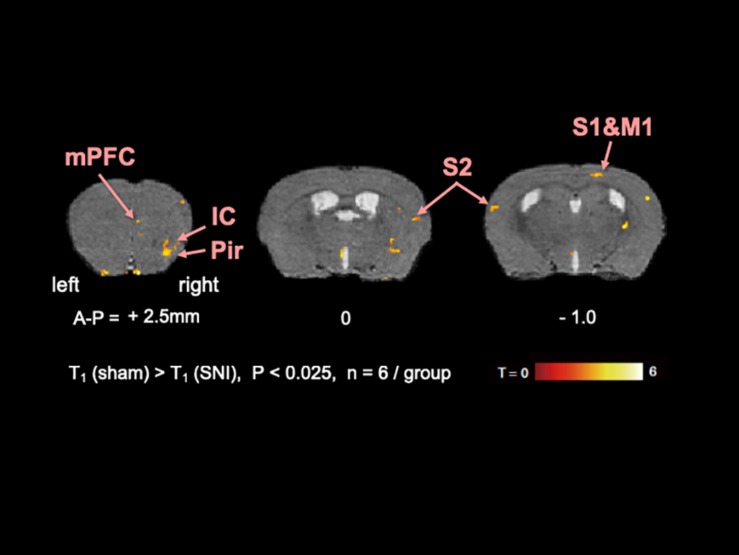
The active regions in the cortex in SNI mice compared with sham mice analyzed by AIM-MRI. Regions with significant shortening of T1 in SNI mice are indicated by the pseudo-colored regions over the T2-enhanced brain image template in coronal planes (*n* = 6 for sham, *n* = 6 for SNI). The active regions in the cortex are defined in text: piriform cortex (Pir), insula cortex (IC), and secondary somatosensory area (S2). The distance from bregma in mm is shown at the bottom. T, *t*-value; P, *p*-values.

To elucidate the activated brain regions in neuropathic pain, qAIM-MRI was conducted on SNI and sham-operated groups of mice. To visualize regions with significantly elevated activity, the voxels with significant T_1_-shortening in SNI mice (*n* = 6) in comparison to sham-operated mice (*n* = 6) were defined as those with *p*-values below 0.025 by SPM analysis (Figures 2, 3). Within the limbic structure, the central amygdala (CeA), nucleus accumbens (NAc), caudate putamen (CPu), globus pallidum (GP), posterior cingulate cortex (PCC), and ventral posterolateral nucleus of thalamus (VPL) showed significant shortening of T_1_ (Figure 2). In the cortex, the regions showing significant T_1_ shortening were observed in the secondary somatosensory cortex (S2), sensory-motor cortex (S1, M1, and M2), and piriform cortex (Pir), and insular cortex (IC) (Figure 3). None of the brain regions exhibited decreased activity under chronic pain. The *t*-values and *p*-values resulting from the Student’s *t*-test and the brain coordinates are indicated in Table 1. To confirm the variability across animals, we conducted Mann-Whitney *U*-test and bootstrap analysis of the mean T_1_ value in each ROI. Significant shortening of T_1_ values were detected in NAc (Sham; 2.35 ± 0.04, SNI; 2.16 ± 0.03, *p* < 0.05), CPu (Sham; 2.50 ± 0.06, SNI; 2.35 ± 0.02, *p* < 0.01), GP (Sham; 2.23 ± 0.04, SNI; 2.09 ± 0.02, *p* < 0.05), VPL (Sham; 2.20 ± 0.03, SNI; 2.06 ± 0.02, *p* < 0.005), Pir (Sham; 2.04 ± 0.04, SNI; 1.86 ± 0.04, *p* < 0.05), right S2 (Sham; 2.52 ± 0.03, SNI; 2.41 ± 0.03, *p* < 0.05), and left S2 (Sham; 2.67 ± 0.04, SNI; 2.51 ± 0.04, *p* < 0.05).

**TABLE 1:** Coordinates, statistical values, and T1 value of the active region in SNI compared with sham mice (*n* = 6) analyzed by AIM-MRI.

	**Coordinate (mm)**			**Sham**	**SNI**	
**Region**	**M-L**	**D-V**	**A-P**	**T_1_ (s)**	**T_1_ (s)**	***P***
**Limbic structure**						
Right NAc	–2.11	3.48	2.0	2.35	2.16	<0.005
Right CeA	–2.62	3.31	0.3	2.30	2.13	<0.005
Left PCC	0.21	7.26	–1.5	2.36	2.22	<0.005
**Basal ganglia**						
Right CPu	–2.79	5.71	0.8	2.50	2.35	<0.005
Right GP	–2.62	3.82	–0.3	2.23	2.09	<0.01
Right VPL	–2.79	4.51	–1.3	2.20	2.13	<0.005
**Cortex**						
Right Pir	–2.02	3.57	2.5	2.04	1.86	<0.005
Right IC	–2.02	4.25	2.3	2.38	2.29	<0.025
Right S2	–3.91	5.11	0.3	2.52	2.41	<0.005
Left S2	4.51	5.63	–0.8	2.67	2.51	<0.005

*M-L, medial – lateral (mm); D-V, dosal – ventral (mm); A-P, anterior – posterior (mm). The active regions are defined in the table, nucleus accumbens (NAc), caudate-putamen (CPu), central amygdala (CeA), globus pallidus (GP), ventral posterolateral nucleus of thalamus (VPL), posterior cingulate cortex (PCC), piriform cortex (Pir), insula cortex (IC), secondary somatosensory area (S2), sensory-motor cortex (S1, M1, and M2). *t* and *p*: *t*-values and *p*-values of Student’s *t*-test, respectively.*

## Discussion

The present results indicate that the neuropathic pain mouse model experienced an increase of resting-state brain activity and that qAIM-MRI can successfully distinguish neural activity in both the neuropathic pain mouse model and normal control mice.

The objective evaluation of pain is particularly important in basic and clinical research. The most widely used methods for pain assessment are behavioral paradigms such as the tail flick or the hot plate test, which can measure the function of the spinal cord and brain stem but not the cerebral cortex (Vierck et al., 2008). Instead of behavioral assessments, brain imaging is a putative complementary evaluation method for animal studies on pain. Several brain imaging techniques have been used to examine various types of pain models while the animal is under anesthesia (Hess et al., 2007; Seminowicz et al., 2009, 2012; Upadhyay et al., 2013). Because pain perception and related behaviors require consciousness and awareness, assessment of brain activity in awake animals is critical for accurate identification of steady-state brain activity (Chang et al., 2017). The qAIM-MRI method enables quantitative neural activity mapping of an awake animal (Kikuta et al., 2015). In the present study, we injected MnCl_2_ intraperitoneally without anesthesia. The Mn^2+^ is taken up into activated neurons while the animal is awake and thus becomes a marker for activity, making it possible to determine previous neuronal activity in awake and free moving animals (Tambalo et al., 2009). As such, the animals could still be anesthetized during MRI for visualization of brain activity. The qAIM-MRI method used in this study enables quantitative neuronal activity mapping over the entire brain and can help characterize changes to brain activity in neuropathic pain.

The present results indicate that the neuropathic pain mouse model have increased activity in several brain regions, including the cortex, limbic system, and basal ganglia (BG). The same brain regions activated in the SNI mice have also been reported in fMRI and PET studies on humans (Peyron et al., 2000; Apkarian et al., 2005), and have been proposed to be the “pain matrix” from brain imaging analysis. The pain matrix includes the primary somatosensory cortex (S1), secondary somatosensory cortex (S2), anterior cingulate cortex (ACC), IC, mPFC, thalamus, amygdala, BG, periaqueductal gray matter (PAG), and cerebellum (Peyron et al., 2000; Apkarian et al., 2005). These brain regions are also activated by nociceptive stimuli in chronic pain patients (Treede et al., 1999; Bosma et al., 2017). There are several reasons behind excluding measurement from the PAG and cerebellum in this study. If data from more slices, including the cerebellum, are acquired, the MRI measurement time increases, which may affect the animal condition. Moreover, since our major focus in this study was to explore the function of the frontal part of cerebral cortex in pain processing, the hind brain regions such as PAG and cerebellum were not evaluated. Further studies are required to reveal the activation of the PAG and cerebellum under chronic pain.

There are several reports wherein fMRI and PET were used to perform whole brain imaging to study pain perception in rodents (Thompson and Bushnell, 2012). Although most studies investigated brain activity after nociceptive stimulation in anesthetized animals, the activated brain regions correlated with the results from human brain imaging studies (Thompson and Bushnell, 2012). In the neuropathic pain model, brain activity in the somatosensory area, cingulate cortex, and thalamus regions are increased after nociceptive stimulation both in anesthetized and awake animals (Baliki et al., 2012; Thompson et al., 2014; Hubbard et al., 2015; Komaki et al., 2016). The resting-state brain activity in neuropathic pain models have also been studied using the [^18^F]fluorodeoxyglucose (FDG micro-PET) method. Brain regions with increased metabolism were primarily located in prefrontal-limbic-brainstem networks, which engage in cognitive and emotional modulation of pain (Kim et al., 2014). In the current study, we also observed increased activation of the prefrontal-limbic-basal ganglia network during resting-state brain activity in SNI mice using qAIM-MRI. Since the qAIM-MRI method directly determines the neuronal activity in freely moving animals, our present results provide comprehensive evidence that the regions in the nervous system related to emotion might be activated under neuropathic pain.

Recently, AIM-MRI studies were performed in SNI rat and the monosodium iodoacetate (MIA) model rat (Devonshire et al., 2017; Chao et al., 2018). Mn^2+^ accumulation in the ventral tegmental area (VTA), right CeA, and left cingulate was negatively correlated with pain response in the MIA model rat (Devonshire et al., 2017). The ipsilateral anterior insular cortex was activated in freely moving SNI rats 1 day after surgery using manganese-enhanced MRI (MEMRI) (Chao et al., 2018). Somatosensory cortex S1, cingulate cortex, and insular cortex were also activated 8 days after SNI surgery. In this study, we observed the activation of several limbic structures in SNI mice 14 days after surgery. Since the duration of chronic pain might differentially affect neural plasticity in each brain region, the functional changes across brain regions might be widespread upon prolongation of chronic pain.

The advantage of qAIM-MRI lies in its ability to measure the absolute T_1_ value, as opposed to several studies using MEMRI that measures the signal intensities obtained from T_1_-weighted images (Chao et al., 2018). T_1_-weighted images are quantified by signal intensities that provide relative values, and therefore can be unreliable for comparisons between animals. The absolute T_1_ value, however, can be used to quantify the Mn^2+^ concentration. Therefore, our present results reflect absolute neural activation under chronic pain.

It must be noted that Mn^2+^ itself could alter neuronal activity. In the present study, MnCl_2_ ⋅ 4H_2_O was intraperitoneally administered at a dose of 0.2 mmol/kg (39.6 mg/kg), whereas Chao et al. (2018) administered a dose of 75 mg/kg intravenously. Kikuta et al. (2015) reported that the Mn^2+^ concentrations in the ventricular regions of mice after intraperitoneal administration at a dose of 0.2 mmol/kg are less than 80 μM. The serum Mn^2+^ concentration after intravenous administration at a dose of 75 mg/kg would be 5.86 mM, because the blood volume in rat weighing 400 g would be 24.77 ml (Lee and Blaufox, 1985). Since the concentrations of Mn^2+^ over 200 μM could alter neuronal activity (Kostial et al., 1974; Hackett, 1976; Bagust and Kerkut, 1980), intravenous administration of MnCl_2_ at a dose of 75 mg/kg seems very high to evaluate the brain activity by MEMRI.

There are a couple of limitations and caveats of the qAIM-MRI techniques. The voltage-gated calcium channels (VDCCs) are expressed in neurons and astrocytes (MacVicar and Tse, 1988; Burgos et al., 2007). The ionotropic glutamate receptors, including N-methyl-D-aspartate (NMDA) receptors, that Mn^2+^ can penetrate through, are also expressed in astrocytes. Since qAIM-MRI utilizes Mn^2+^ as a contrast agent, excluding the influence of Mn^2+^ uptake in astrocytes is challenging. Astrocytes, however, can respond to presynaptic neurotransmitter release, and Mn^2+^ uptake could occur in response to neuronal activity (Kikuta et al., 2015). Therefore, Mn^2+^ accumulation in astrocytes may correlate with the activity of adjacent neurons.

## Conclusion

In conclusion, changes to neural activity in areas that process pain cognition and emotion contribute to the chronification of neuropathic pain.

## Ethics Statement

All animal studies were approved by the Animal Care Committee of the Graduate School of Pharmaceutical Sciences, Nagoya City University and the Tohoku University Committee for Animal Experiments. Experiments were conducted in accordance to the guidelines of the National Institute of Health and the Japanese Pharmacological Society.

## Author Contributions

CI, HT, SK, OO, MOs, and MOh performed the experiments and data analysis. MOs and MOh calculated the statistical significance and wrote the manuscript. KS and KK supervised the study.

## Conflict of Interest

The authors declare that the research was conducted in the absence of any commercial or financial relationships that could be construed as a potential conflict of interest.

## Footnotes

1 http://mouse.brain-map.org/
